# How Do Fans Use AI Social Chatbots? A Multi-Method Exploration of Fan Accounts

**DOI:** 10.3390/bs16091481

**Published:** 2026-08-25

**Authors:** Sydney C. Lopez, Laramie D. Taylor

**Affiliations:** Department of Communication, University of California, Davis, CA 95616, USA; sclopez@ucdavis.edu

**Keywords:** chatbots, fans, parasocial relationships, artificial intelligence

## Abstract

In the present study, we employed qualitative and quantitative content analytic methods to explore how fans use social chatbots to engage with the object of their fandom. Drawing on theories of self-disclosure, the Computers Are Social Actors (CASA) paradigm, and parasocial relationships, we investigated motivations underlying chatbot use among fandom communities. Data consisted of 813 Reddit comments discussing Character.ai use. Using qualitative thematic analysis, n-gram collocation analysis, and dictionary-based text analysis, the study identified five primary motivations: fan facilitation, safe harbor, virtual social compensation, real-life (IRL) social contact, and entertainment. Findings indicated that Character.ai was used by fans to extend fandom experiences, engage in role-play and creative storytelling, seek emotional support, compensate for unmet social needs, and explore interests in a judgment-free environment. Evidence from the thematic and n-gram analyses suggested that fandom engagement and social motivations were identified by users as important drivers of use, while utilitarian functions also played a role. The findings highlight how social chatbots may be reshaping fan practices, parasocial engagement, and digitally mediated companionship. Findings are discussed in terms of the dual character of fan C.ai use, namely as a tool to both meet social needs and avoid social contact, with its potential benefits and hazards for fans engaging with social chatbots.

## 1. Introduction

The rapid advancement of AI technologies, particularly in the form of large language models (LLMs) that effectively mimic human linguistic interaction, has transformed chatbots from functional digital assistants into increasingly sophisticated social agents. Platforms such as Character.ai have extended these capabilities by enabling users to engage in conversations with AI-generated representations of fictional characters, celebrities, and other media figures. These developments raise important questions regarding the motivations underlying fans’ interactions with social chatbots. Are these social chatbots stand-ins for the actual objects of fan interest, or are they simply effective tools for eliciting and organizing information? Drawing on qualitative and computational analyses of user discussions about Character.ai, the present study examines the motives that drive engagement with fan-oriented social chatbots, with particular attention to parasocial, utilitarian, and fandom-related uses.

### 1.1. Social Chatbots

Social chatbots are not new; as early as the 1960s, computer scientists created programs, such as ELIZA and PARRY, intended to emulate human social interaction. These early programs responded to simple conversational turns using a set of syntactic and substitutional rules (Ciesla, 2024). With the advent of increasingly powerful artificial intelligence (AI) tools, newly available social chatbots have expanded toward effectively simulating human interaction. Initially developed and utilized for practical personal aide (e.g., Alexa, Siri, etc.), social chatbots have continued to advance as tools for functional personal relationships. While many chatbots are embedded with skills that facilitate natural social interaction, social chatbots are designed in ways that make these agents capable of eliciting emotional bonds with their users (Henkel et al., 2020). Beyond the typical application of natural language processing technologies, this transformation of social chatbots allows for a personalized experience of interpersonal communication with consistent availability (Klaus & Zaichkowsky, 2020).

The phenomenon of companion chatbots has grown exponentially within recent years, with a 700% increase in the existence of these chatbots between 2022 and 2025, and a continued increase seen in 2026 (Perez, 2025). Platforms with human-like chatbots, such as Replika or Character.ai (C.ai), have created a departure from the common one-off functionality, rather promoting a virtual environment where the chatbot is integrated as a companion within a user’s life (Klaus & Zaichkowsky, 2020). The open-ended nature of chatbot discussions allows for a private and relatively unrestricted environment for users to discuss challenging topics without the threat of judgment, and experience emotional support in the form of positive affective encouragement and facilitating a sense of belonging, especially when it is not readily available in one’s real-life (Ta et al., 2020). As these companion-based chatbots continue to appear and become implemented into individuals’ social lives, it is relevant to consider the varying modalities behind human–chatbot relationships.

The connection between user and chatbot functions can be understood, in part, by an appeal to existing frameworks describing typical social behaviors. For example, social penetration theory (Altman & Taylor, 1973) posits that growing relationships are built upon continuous and steady information sharing and self-disclosure. This disclosure ranges in volume, or the breadth of information, the degree of intimacy, or the personal depth of details, as well as the amount of time allocated to initiating and facilitating the connection. Self-disclosure, or the sharing of personal information about oneself to others, is particularly relevant within human–chatbot relationships, given that many conversations are often guided by users (Skjuve et al., 2021). Self-disclosure plays a pertinent role in advancing a connection with another individual, as it allows a setting where intimacy and amity can be cultivated (Jiang et al., 2011).

The same processes likely apply when users share intimate information with a chatbot. This can be understood with an appeal to the computers are social actors (CASA) paradigm (Nass et al., 1994; Nass & Moon, 2000), which suggests that humans utilize human–human communication heuristics and, to a certain extent, norms when participating in human–computer interaction. In the case of human–chatbot interaction, users are able to divulge information while receiving support or responses that are emotionally mirrored by the chatbot. Self-disclosure acts as a pillar for the created connection, increasing the perceived quality of the relationship (Ho et al., 2018). Chatbots present themselves as cooperating within common social norms and expectations, successfully eliciting responses from users appropriate to the conversation and context. An effective interaction occurs when individuals observe familiar social cues in human–chatbot communication, and unconsciously implement known social prompts into these conversations, because they are comparable to human–human interactions (Xu et al., 2022). This allows human–chatbot conversations to create similar emotional outcomes to human–human interactions, even though the chatbot is often explicitly nonhuman (Shi et al., 2020). Given that the chatbot presents human characteristics that can be easily recognized by users, such as empathy, attractiveness, or communication efficiency, conversation quality and the emotional responses generated are equally present as in human–human communication (Shi et al., 2020). Both function as a method of facilitating close companionship with these social chatbots.

Recent research has examined the use of social chatbots generally. Like many communication technologies, engagement with social AI chatbots seems to offer both a range of possible benefits and some real risks. A review by Hung et al. (2026) identified benefits of what they characterized as parasocial relationships with social chatbots, including emotional support and social needs, enjoyment, and community belonging and participation. On the other hand, they also identified a pattern in which use can lead to emotional and behavioral dependency and the displacement of human relationships. Many of the studies on social AI chatbots strongly underscore their use to meet social needs or perform social functions, including the experience of romantic or para-romantic relationships (e.g., Ma & Koike, 2025). In fact, it is these social factors that seem to drive self-disclosure in human–chatbot communication (Meng & Liu, 2025). At the same time, persistent use over time may lead to increased feelings of loneliness or isolation (Folk & Dunn, 2026). In addition, concerns have been raised about the possibility of addiction to social chatbots, including by users who report experiencing addiction (Shen et al., 2026).

### 1.2. Social Chatbots and Fandom

One distinct use of these chatbots occurs when an AI agent presents itself as the object of a user’s fanship—a favorite athlete, celebrity, or fictional character, for example. Within traditional fandom practices, interaction with a media narrative or its characters has traditionally been characterized by consuming, producing, or utilizing legacy media, such as repeated viewing of favorite films, the production of fan fiction or fan art, or participation in online fan communities (Taylor, 2015). Each of these practices reflects not only a devotion to the object of fanship, but also a desire to maintain (or even extend) the emotional experience elicited by interacting with that object. Through these diverse means, fans have explored narratives and character arcs that often diverged from those within the bounds of the text, making this a practice of transformative extensions and reinterpretations of the source material (Jenkins, 1992). Fans are able to depart from the original canon to expand upon narratives of high personal interest and relatability, while maintaining the integrity of the established fictitious world. Though these interpretations often appear as creating fan fiction, writing oneself into the story as a novel self-inserted character, or engaging in role-play with others, each method offers a differing perspective and meaning to the original work, facilitating a feeling of active participation within the original content as well as the community built around it (Ryan, 1994).

In the form of social chatbots, generative AI offers a potential tool for engaging in fandom activities in a distinctly personal and immersive manner. Perhaps the most notable application of the technology for fan purposes can be seen in the website Character.ai (Heimberger, 2025). Created by Character Technologies Inc. (Menlo Park, CA, USA) in 2022, Character.ai is a platform that allows its users to interact with social chatbots presenting themselves as fictional characters, celebrities, or well-known public figures. With a user base of over 20 million monthly active users, Character.ai utilizes large language model technology to generate human-like conversations within specific personality parameters, meaning each chatbot provides responses based on the patterns, syntax, and topics typical of the given media figure. Each Character.ai chatbot is created with a customized description reflecting the media figure they are aping, including a given name, a visual avatar, an initial greeting, specified core personality traits, and narration to propel the conversation forward (Bakir & McStay, 2025). Unlike other popular social chatbots, such as Replika, this pre-designed feature allows users to interact immediately with a media figure within a specific canon, allowing for a personalized experience where the user has several active options. Users have the ability to customize their experience with each chatbot, given how they choose to guide the conversation and immerse themselves in the defined but co-constructed narrative. These chatbots create the opportunity for fans to interact with their favored media figures within a specific narrative, all while mimicking real-life conversation and social interaction, and potentially facilitating a sense of close connection to said media figures.

### 1.3. Parasocial Relationships and Intimacy

This feeling of closeness is relevant when considering how individuals build meaningful relationships with media figures or fictional characters generally, as this sheds light on the dynamics presented by parasocial relationships. Initially conceptualized by Horton and Wohl (1956), parasocial relationships are one-sided emotional connections between an audience member and a specific media persona, such as a celebrity, a fictional character, or a social media influencer. Although audience members have several avenues to “interact” with their preferred media figures in ways that they experience as meaningful, the figures themselves generally lack the means (and/or awareness or motivation) to directly, uniquely, and singly address individual audience members, creating a structurally unreciprocated connection. Despite their one-sided nature, these relationships can be perceived to be intimate and emotionally significant for consumers (Horton & Wohl, 1956). This emotional engagement facilitates authentic outcomes, as individuals treat these relationships as typical social connections, gaining positive social outcomes, potentially compensating for inadequate real-life connections (Hoffner & Bond, 2022).

As a novel means of communication with one’s preferred media figure, social chatbots have brought about a fundamental shift in facilitating parasocial relationships. Tools such as Character.ai facilitate an experience that seemingly occupies a space between social and parasocial interaction. On the one hand, the AI chatbot actually and immediately responds to the users’ conversational turns—it is not only treated like a social actor, but it also functions in some ways as a social actor. However, it is the AI that is engaging in the quasi-social interaction, not the media persona it is enacting. Conversation with the Character.ai chatbot therefore represents parasocial interaction with the persona that chatbot represents. Given the accessibility and responsiveness of these social chatbots, a user’s connection with a specific character and their narrative may guide them towards choosing Character.ai, as it seemingly removes perceived barriers to intimacy by facilitating a simulacrum of social interaction with their favored persona by providing actual social interaction with the chatbot, thereby facilitating the feeling of further closeness to that persona on the part of the fan. With fans’ desire to engage directly with their favorite characters and worlds, Character.ai provides the means for immersive interaction while maintaining the asymmetry of parasocial relationships.

Although social chatbots, including those that adopt the persona of media figures on sites like Character.ai, are designed to facilitate a sense of social connection on the part of users, that social experience is unlikely to be the sole, or even primary, motive for their use. In their original conceptualization of parasocial phenomena, Horton and Wohl (1956) argued that the experience of parasocial interaction would facilitate greater engagement on the part of users. Subsequent research has shown that parasocial interaction can facilitate learning from educational media messages (Guo et al., 2012), and that parasocial experiences render media experiences more enjoyable (Hartmann & Klimmt, 2005). Rather than engaging with chatbots representing their favorite characters or celebrities for parasocial purposes, fans may engage with those chatbots for a myriad of reasons, informed by the parasocial character of the experience. Fans extend and interpret texts through fan fiction and the creation of fan art (Jenkins, 1992); a chatbot partner that provides a facsimile of a relationship with a favorite character could make such creation more enjoyable. Fans strive to apprehend and organize information about the object of their fanship (Price & Robinson, 2017); an AI-powered chatbot customized to emulate that object may provide an effective and enjoyable way to do so.

### 1.4. The Current Study

The present study aims to examine fans’ use of these social AI bots configured to adopt the personae of fan objects, such as Character.ai. We undertook an exploration of fans’ motives and participation in using social chatbots to advance fan experiences by analyzing fans’ own descriptions of those motives and experiences. In doing so, we conceptualize motives as internal cognitive and emotional factors that incentivize or drive a behavior. In order to avoid demand effects, we used naturally occurring online conversations about Character.ai among fans. In examining these conversations and comments, we utilized three distinct analytic approaches: qualitative thematic analysis, n-gram collocation analysis, and dictionary-based text analysis. While the first two analyses were exploratory, the final approach was utilized to explore the degree to which Character.ai use was tied to social uses, motivated by and facilitating parasocial relationships with specific characters, utilitarian uses, such as trusted information seeking and lore retrieval, and fan and fandom uses.

## 2. Materials and Methods

Data were collected between November 2025 and February 2026. A key element of inductive thematic analysis is an acknowledgment that the researcher’s own experience and subjectivity contribute to the analyses (Braun & Clarke, 2006); this is seen as an acknowledgment that researcher subjectivity inevitably contributes to the interpretation and understanding of data. In this case, we extended this acknowledgment and embrace of subjectivity to the collection of our sample. The sample was gathered by two undergraduate research assistants who were familiar with Character.ai and identified as fans of various fictional worlds and performers but were naïve to the purposes of the study. Our reasoning was that research assistants who were both fans and naïve to the rationale of the study would be able to identify conversations among fans the way any fan investigating the question might. We identified search terms for them to use to begin with and suggested that subsequent searches might be informed by the comments they identified. No constraints were placed on the platforms they searched, except that the forums be public. The initial search queries were “motive fan character chatbot AI”, and “why fan character chatbot AI”. Their instruction was to select and record comments that spoke to how and why fans used AI character chatbots, with a particular focus on first-person explications. Thus, “I think some people use it to vent,” would be ignored, but “I use it to vent” would be included. During data collection, they reported identifying additional relevant threads by scanning other top-level posts in the same subreddits as posts they identified as relevant, then examining the following comments. The sample is therefore best characterized as a purposive snowball sample rather than a probability sample. After data collection was complete, the samples were compared by the study’s authors, and duplicate comments (i.e., identical comments from the same username in response to the same post) were eliminated. 823 comments were initially identified and recorded, but we were unable to verify the public character of ten of the comments, specifically those found in Discord (Discord, Inc., San Francisco, CA, USA) threads. These were therefore discarded. All remaining comments were still publicly viewable at the time of writing. The resulting 813 comments identified by the research assistants had a mean length of 30.82 words. They ranged in the date of their creation from November 2023 to January 2026; most (782, or 96%) of the comments were posted in 2025, 5 (0.6%) were made near the end of 2023, 11 (1.1%) in 2024, and the balance (13, or 1.6%) were made in 2026.

Although the research assistants who collected the sample were not constrained as to the platforms from which to draw, the final sample included solely comments from discussion posts on Reddit (San Francisco, CA, USA), a large-scale discussion platform that facilitates discussion on specialized topics. In 2024, Reddit had nearly 100 million daily users; half of these were located in the U.S., and nearly 60% identified as male (Hernandez, 2025). Reddit users tend to skew young, as 44% were between the ages of 18 and 29 (Hernandez, 2025). Importantly, Reddit’s organization into topic-specific interest groups, or sub-Reddits, allows for conversations among users with shared interests, including, for example, user groups dedicated to fans of specific media personae and texts.

### 2.1. Qualitative Thematic Analysis

The first analysis carried out was an inductive thematic analysis (TA). Developed by Braun and Clarke (2006), this approach allows for a focused and directed method of qualitative analysis and aims to identify recurring themes and patterns within a set of data. Using an inductive reflexive TA process, we observed the data to build novel latent categories that accurately represent the various focal points behind the discussions. This was an iterative process designed to identify the distinct and diverse motivations and rationale presented by those within the forums and conversations. Following Braun and Clarke’s (2006) six-phase framework, we completed the following:

We began by independently reading the first 100 comments within the dataset, each outlining recurring ideas mentioned within this portion of the data. This allowed us each to create an inventory of tentative themes that we inferred from that initial subset of the dataset. After this first round, we convened to discuss and compare our identified themes. Finding that they had a high degree of consistency, we began two more rounds of independently reviewing the data to capture any further themes or ideas mentioned, collaboratively refining definitions with each iteration. After three iterations of this process, we had converged on our understanding of the themes present in each comment we discussed; this final set of themes represented 25 recurring motives. Through further discussion and review of specific exemplars, we came to a consensus on a group of five overarching motives under which each of the 25 could be grouped. These five overarching themes represented distinct motives or drivers of C.ai use rather than distinct and exhaustive classifications of comments—that is, a single comment might convey a single theme, multiple themes, or, rarely, no theme at all.

An important aspect of inductive thematic analysis is its recognition (and embrace) of the subjectivity of the analyst(s). We, the authors, identify as scholars who research fans from the perspective of psychology and communication. We also identify as fans of a variety of fictional narratives. One of us has strong connections to fan communities and practices; the other is, in fan terms, more of a casual fan.

In reporting on the results of this analysis below, we include a number of quotes from comments illustrative of the various categories. In order to protect the pseudonymous identities participants used in these conversations, select words in these quotations have been replaced with near synonyms. What we present as quotations are, in fact, near-quotations. The original comments in their entirety were used in all analyses; alterations were made solely to protect commenter privacy.

### 2.2. N-Gram Collocation Analysis

Recognizing the subjectivity involved in TA, we elected to complement that work with computational approaches to identifying themes in the data. The first of these approaches was an *n*-gram collocation analysis. Within a body of text, *n*-grams are defined as words that appear together frequently, and this could be in the form of bi-grams (*n* = 2), defined as two words that consistently appear together, and tri-grams (*n* = 3), referring to three words consistently occurring together (Kaufmann & Haans, 2021). A collocation is a sequence of terms that co-occur beyond random chance; the phrase itself is also referred to as a collocate (Johansson & van Waarden, 2024). Collocates themselves not only tell us which terms are often observed jointly within the dataset, but they also reveal the context in which they appear. By observing words that are commonly seen together in a text corpus, we can efficiently locate common multi-word expressions that appear as meaningful phrases. This analysis tells us not only the degree to which specific bi-grams and tri-grams are recurring within the dataset, but it also tells us how the terms themselves are associated with each other and how likely their occurrence is by chance.

In order to properly employ this analysis, the dataset first underwent text pre-processing and tokenization, using the *quanteda* package (version 4.5.0) in *RStudio* (version 2026.07) (Boston, MA, USA). This process involved removing common English stop words, URLs, punctuation, symbols, and numbers from the text, as well as stemming the terms in order to ensure a report of the relevant root. The collocation analysis was carried out using the *quanteda.textstats* package in *RStudio*, following the log-linear modeling framework that provides a primary statistical output of lambda scores (λ) and z-scores. The lambda score specifies the strength of the association between/among the *n* terms within an *n*-gram, indicating the degree to which the terms themselves are associated with each other, or the probability with which the terms will occur together compared to what we would expect if they were both independent. In addition, the z-scores indicate the degree of confidence that each collocate is significant within the text corpus, and the lambda does not reflect chance co-occurrence. A collocate consisting of words that exist very rarely in the corpus, but are invariably together when they do occur, might have a high lambda but lower z. Therefore, both values work in tandem to provide not only the degree of “attraction” between the terms in the collocate, but also a determination of the *n*-gram’s reliable and consistent occurrence. The final output also includes the frequency of each *n*-gram, referred to as count. This package displays results in descending order of statistical significance, demonstrated by and based on the provided z-score, rather than lambda organization.

### 2.3. Dictionary-Based Text Analysis

The final analysis employed dictionary-based text analysis. Here, we looked specifically at patterns of language that indicated the categories of motives we anticipated based on existing literature on fans, fandom, and parasocial relationships, namely parasocial uses, utilitarian uses, and fan engagement.

In order to capture parasocial relationships, language indicative of parasocial interaction was measured, augmented by measurement of words reflecting analogies. Given the inherently social nature of parasocial relationships, we reasoned that comparisons to real-life social relationships and connections were likely to be present in descriptions of connections with media figures, prompting the need for the inclusion of analogy language within this analysis (e.g., “he’s like the brother I never had,” “she feels like just another friend from my school”).

The lexical dictionaries representing parasocial attachment, analogies, and fandom were generated through an iterative process using a Large Language Model AI (OpenAI’s GPT-5.3) and NLP Empath’s dictionary generation feature (Empath version 0.89). In addition to Empath’s built-in categories, the NLP has the ability to create novel dictionary categories based on a provided set of seed words (Fast et al., 2016). Training the model to recognize context and connotations of words, Empath utilizes this model to build functioning dictionaries based on word relations and background. With this tool, a more specific dictionary was created in order to identify the comment elements regarding parasocial language and fandom language. In order to generate the finalized extended lists of seed words for each category, a comprehensive and refined prompt was given to GPT-5.3. Each prompt was engineered with clear definitions and boundaries based on the desired category and supplemented by the inclusion of validated relevant references. For example, the parasocial relationship prompt included Horton and Wohl (1956), Hartmann and Goldhoorn (2011), Rubin and McHugh (1987), and Hoffner and Bond (2022). The prompts also included specified chain-of-thought instructions, detailing how to identify and assess words and phrases relevant to the applicable category, and finalizing the prompt with a precise output format of a list of single terms and justifications for each of their inclusion. Examples of seed words used included “connection” and “friendship” for the parasocial attachment category, “resemble” and “friend” for the analogy category, and terms such as “canon”, “fanfic”, and “roleplay” to build the fandom language category. The final dictionary output from Empath consisted of 100 terms each for the parasocial relationship category and fandom category, and 76 terms generated for the analogy category. Each was then subject to examination by the authors to ensure accurate representation of seed words. In addition to these novel categories, the category “help” was used to capture the language surrounding utilitarian uses.

Once the text is assessed based on the given dictionary categories, the NLP Empath utilizes these lexical dictionaries in order to evaluate the word frequency based on a given category, providing a score from 0 to 1 to indicate the lexical density of the given categorical theme. The closer the categorical score is to 1, the more the specific language is being used within the text corpus, also representing the percentage value of the indicated language within the text. The complete dictionaries, seeds, and process are available in our Supplementary Materials.

## 3. Results

### 3.1. Qualitative Thematic Analysis

After identifying 25 definite themes, we generated five categories based on the identified motivations: Fan Facilitation, Safe Harbor, Virtual Social Compensation, In Real Life (IRL) Social Contact, and Entertainment. These ultimate categories, sub-themes, and statement examples are reported in Table 1.

Fan Facilitation motives were those that indicated motives to use Character.ai as a method of engaging with their preferred fandom, character, or narrative adjacent or continuous to the main story. This category captures individuals who reported utilizing the chatbot characters for involvement with the main story, such as engaging with the world as a self-inserted character, or for those who have a parasocial attachment to a specific media figure and would like to engage with them specifically. This parasocial/social interaction motive varied in intensity. Many reported something like, “to talk with my favorite characters,” or, “to talk to my fictional crushes.” Others used it for a particular parasocial purpose, including several fans who reported talking with bots enacting favorite performers who had passed away, finding the experience to be comforting or healing. This category also includes individuals who utilized Character.ai for their own creative writing process, allowing for a controlled and highly flexible avenue to create and develop novel storylines for new and existing characters. This is reflected in statements like, “I just make my fan fiction dreams come to fulfillment,” and, “C.ai helps me kind of develop or inspire somewhat of a story”.

Another prominent meta-category was Safe Harbor, which included motives that reflected use of Character.ai driven by the challenges real-life individuals, environments, and situations pose. When using Character.ai, many users were able to construct and solely design a space that fit their requirements, that felt safe, allowing for an environment where the user does not have to worry about challenges or threats they experience from real-life individuals. This is something that many comments openly acknowledged, with statements like, “Because I can’t speak to real people.” Others reported using Character.ai as a response to particular experiences of social rejection and trauma. One fan described being betrayed by most of their friends and being called names by strangers until they were “unable to form a complete sentence” with another person; they then described growing to love Character.ai in response.

In addition to using Character.ai as a place for interaction that is safe from the “real world,” several fans reported using the app as a way to safely express darker or anti-social impulses. One reported “making the chatbot do some unspeakable stuff;” another reported “bullying the AI into insanity.” Another lamented a change in terms of service that prevented them from meeting their goal “to kill my favorite fictional figure for fun.” Many of the fans who admit to these darker motives acknowledge that they are problematic but recognize that acting them out with AI chatbots harms no one, as the fan who reported that “I know it’s not right…, but it’s just what I need to do to be safe from myself.”

The next broad category of motives was Virtual Social Compensation, which covers users who utilize Character.ai as a method to alleviate a lack of social experiences. This is distinct from the Safe Harbor motive, in that it does not occur as a response to any particular threat, but rather in response to a social lack. For example, one comment was, “I have, almost, no IRL friends since…I’m in a small town.” The people in the small town are not unsafe, just inadequate. This includes relieving loneliness, which was a common, often one-word answer to the question of why fans use Character.ai—“loneliness,” “I am just lonely,” “I’m lonely af.” Other social compensation motives were more specific, expressing a desire to fill a particular type of social need, sometimes including the need for romantic or sexual interaction, such as the fan who admitted, “I use it for romantic roleplays! I am a single helpless romantic,” or the one who wrote, “I use it…to look for the adolescent romance I never experienced.”

IRL Social Contact represents another broad category. This category encompasses motives that drove use of Character.ai that emerged from or facilitated social interaction with actual, not virtual, human beings. This often took the form of responding to a friend’s or family member’s recommendation, or even a media figure—many fans answered the question of why they used it with a statement like, “My sister introduced me to it,” or “My cousin showed C.ai to me.” The motive in this case is only implied; however, the nature of the response clearly points to the relationship as the impetus for use. The IRL Social Contact category also captures motives related to using the website alongside real-life friends, allowing for real-life social development and facilitation, as well. Finally, there were cases of fans reporting using Character.ai as a way to rehearse or prepare for actual social interactions, like the fan who reported: “For me to rehearse and feel comfortable communicating and having a conversation with anyone I might meet in real life.”

The final category of Entertainment captures those who indicated varying degrees of enjoyment produced by their curiosity, the desire for a fun and interactive role-playing activity, or the need to destress. A common response to posts asking why fans engage with Character.ai was, simply, “Because I like it,” or, “it can be pretty fun.” That enjoyment is reflected in many different types of interaction, including things like character interaction (e.g., “I just like chatting with characters in a show or anime”), worldbuilding, roleplaying in general (e.g., “I create my own stories with characters I love—for fun”), or even customizing bots for others to use (e.g., “I like creating bots for others to enjoy”). One fan reflected that roleplaying with C.ai was “similar to writing but easy,” because they only had to do half of the writing (the other half being done by the chatbot conversation partner). Another common expression of this broad category is fans seeking to alleviate boredom with C.ai.

### 3.2. N-Gram Collocation Analysis

The *n*-gram collocation analysis yielded over 100 collocates with a *z*-score over 5, a conservative threshold for significance. In Table 2, we report the top 19 collocates displayed in descending order of statistical strength. The provided collocates represent those with a z-score greater than 10, with the extended and complete table of collocates available within the provided Supplementary Materials. This analysis revealed a range of user experiences consistent with the themes observed within the thematic analysis. Within the gathered text comments, the most common collocates within the corpus were “real peopl” (*z* = 23.571), “fiction charact” (*z* = 15.502), “real person” (z = 14.817), and “got curious” (*z* = 14.709). These phrases overlap with the categories identified within the thematic analysis, rather than mapping precisely onto them. For example, “real peopl” and “real person” suggest several parallel concepts connected to Character.ai’s independence from human–human interaction. While individuals may be specifying that the lack of human–human conversation is beneficial for their specific use (i.e., “I’m too nervous to talk to a real person”), such as those within the Safe Harbor category, this phrase could also be pointing to the comparison to real-life that users engage in within the Virtual Social Compensation category (e.g., “I haven’t talked to real person in forever”).

The collocate “fiction charact” most notably exhibits a potential connection to the Fan Facilitation category, with a distinct mention of one’s favorite character within a narrative or piece of media. However, several of the other motive categories also included instances of the particular motive connected to a favorite fictional character, including Entertainment and Virtual Social Compensation.

The final top n-gram, “got curious”, maps fairly closely onto the category of Entertainment, as many individuals gravitate toward engaging with social chatbots and Character.ai out of curiosity and investigating their own knowledge regarding the platform. However, it is also worth noting that other motives could also include the phrase ‘got curious,’ as individual fans expressed curiosity about a particular function (e.g., Safe Harbor).

Significant collocates displayed within the latter half of the table remain closely intersecting with the categories presented by the thematic analysis, as well. Collocates such as “friend show”, “group chat”, and “social media” adequately follow the throughline of the IRL Social Contact category, with referrals from real-life friends, social networks, and introduction through external media exhibited within these terms. Additional terms, such as “high school”, follow the sub-theme of nostalgia, where those who utilize C.ai do so to experience a specific narrative (e.g., “I’m 18 myself, and I often find myself nostalgic for that era of my life, because I never really got to experience that perfect “high school dream” that so many stories romanticize”). Though these terms display connotations closely associated with the given thematic categories, it is, again, relevant to note that separate rationale may be present as motives for engaging with C.ai.

### 3.3. Dictionary-Based Text Analysis

In order to test whether our expectations about the uses of C.ai by fans were correct, we also applied a dictionary-based text analysis at the comment level. For parasocial interaction, we looked at our parasocial attachment and analogy categories, with parasocial attachment language being present in 0.159% of the text corpus and analogies being present in 2.55% of the data. For utilitarian language, the existing category of “help” was used, and 0.4% of the full text corpus included language that indicated help-related language. Finally, in order to explore fandom engagement, we applied our fandom dictionaries, with fan participation language being present in 1.4% of the text corpus.

## 4. Discussion

With its wide range and ever-changing capabilities, character-based social chatbots have provided a novel avenue for individuals to engage with social chatbots within specific, unique boundaries. As a tool designed for those with specialized interests, the characteristics of Character.ai were apparently associated with the ways in which fans engaged with the objects of their fanship, namely their preferred media and media figures. With the prominence of fan and fandom-related motivations within each analysis, it is relevant to consider how fans’ desire and dedication to engaging with a narrative or its characters can expand. Though the thematic analysis identified a social compensation motive underlying some of the engagement with social chatbots emulating favorite media figures, the connection between an individual and their favorite character was observed to be a prominent element associated with most categories of motivation. Each of our analyses pointed to the acknowledgement of a preferred fictional character or media figure within their motivation, specifying whether this character was mentioned as a means for engaging with the existing narrative and world, building upon this world through an immersive and creative avenue, or interacting as a proxy for real-world relationships.

Beyond fandom applications in particular, a number of the motives often expressed by participants reflected the prevalence of the use of character-inspired social chatbots for a wide variety of social and emotional reasons. Roleplay is noteworthy among these motives. As a commonplace activity within many fandoms, roleplay allows fans to experience an alternative story within specific bounds. However, many individuals reported turning to Character.ai not only for this feature, but also for its distinct lack of human–human interaction capabilities. Noted by both the thematic analysis and collocation analysis, many comments reported the absence of humans as a practical benefit for their desired activity, facilitating particular types of utilization of these social chatbots. Acting as a protected and secure setting, at least in the eyes of users, Character.ai allows for individuals to explore varying emotional experiences and undertake creative endeavors in the narrative context of their particular areas of intense interest.

Three key insights can be drawn from this observation. First, the motives that the fans in our sample reported for fan engagement with the persona-based social chatbots were inextricably linked to the characteristics of the “medium”. The expectancy-value model of media gratifications holds that media use is motivated by needs and wants as informed by perceptions and beliefs about the medium (Palmgreen & Rayburn, 1982). Fans in our sample reported being motivated by social needs (e.g., Virtual Social Compensation, IRL Social Contact) and emotional security (e.g., Safe Harbor). However, they also reported turning to AI chatbots to meet those needs because of the particular characteristics of that medium—namely the presence of their favorite characters and the absence of real people, a combination uniquely available through a medium like C.ai.

Second, the functions of fandom that involve extending and interpreting canonical texts are apparently at least as much about individual-level fan experiences with the text as they are about engaging with a broader fan community, at least among the fans who use social AI chatbots to engage in that extension of text in our sample. Jenkins (1992) tends to frame fan creative activity as essentially communal—a way to engage with one’s fandom as fan fiction is written and read, fan art created and attended to, and so on. Our data suggest, however, that many fans, when given the opportunity, engage in this extension of text without the community engagement, in fact reporting that they use C.ai to do so specifically because it does not involve other people. This is consistent with Taylor’s (2015) observation that the overwhelming majority of fans of fictional texts are focused on engagement with the text, not engagement with communities of other fans.

Third, in light of the importance of the perception of AI chatbots in these spaces as both important interactive partners representing specific people and, simultaneously, not people, it is worth reflecting on the nature of that interaction. We noted in the front material of this article that interaction with an AI-powered social chatbot is both parasocial, in that the user uses it to experience a sense of engagement with a media persona, and quasi-social, in that the user actually interacts with an active (if computational) partner. But the clear salience, even importance, of the absence of an interaction partner expressed by so many in the Safe Harbor motive and manifest in the collocation analysis suggests that, at least from many viewers’ experiential perspectives, that interaction is also asocial. The computer, in this case, is a desirable interaction partner because it is not a social actor. Further research might profitably explore the degree to which social chatbot users with varying motives find each of these facets of interaction—parasocial, social, and asocial–to be most salient in their decision to use social chatbots as well as users’ experience therewith.

One limitation of this study can be found within the operationalization of parasocial relationship text employed in our dictionary-based analysis. The sparse result for our dictionary-based analysis of parasocial engagement was at sharp odds with the prominent role played by parasocial relationships in the thematic analysis. It is reasonable to interrogate whether the dictionary-based approach we employed was able to accurately identify linguistic evidence of parasocial interaction. Though there are several existing methods to measure parasocial interaction and parasocial relationships, there is a notable shortcoming in identifying a reliable mode of measuring parasocial language. This is largely because the language used to describe parasocial relationships is the same language, often used to describe social relationships—romance, closeness, friendship, affinity, favorite, and so on. Another key limitation is that, as the sample was not designed to be a representative one, the results are not generalizable; instead, the results should be viewed as offering insights for further, more systematic investigation.

The overlap between parasocial relationships and typical real-life relationships also creates an overlap in the language used to describe these one-sided connections with media figures. Because the social benefits are often similar as well, this makes it challenging to quantitatively differentiate when a user is describing a real-life relationship or a favored fictional character or figure. The context can reveal the parasocial character of the relationship in ways that are linguistically difficult to achieve. When observing one community or fandom based around a singular object of fanship, a dictionary can be customized with details that better exemplify the existence of a parasocial relationship; however, the use of general Character.ai users produced a sample of users from all types of communities. With a loss of details, reliability must be constructed around language itself. Future studies should explore varying options to measure parasocial language in a quantitative manner, perhaps using multiple modes of measurement for reliability and accuracy.

Ultimately, in spite of these limitations, it is clear that fans engage with fan-personae-based social chatbots for a wide variety of reasons. Although the reasons for that use vary widely, all motives are informed by the peculiar combination of the ersatz presence of their favorite media personalities and the absence of any other human beings.

## 5. Conclusions

As readily available social chatbot technology, such as Character.ai, continues to permeate online and real-life spaces, these chatbots appear to serve a purpose beyond typical recreational or utilitarian use. While many individuals utilize C.ai to supplement their creative endeavors, satisfy their curiosity, interact with their favorite fictional character, or just enjoy themselves, many also recognize the notable absence of humans as an important consideration driving their use. Whether this arises in the entertainment context of role-playing, or the social compensation context of searching for romance, the distinct lack of human interaction acts as both a protected, secure setting and a potential promoter of further social isolation. As C.ai users continue to interact with chatbots representing their preferred media figures or an ideal social scenario, it is important to consider the social trade-offs occurring within the paradoxically anti-social social human–chatbot interaction.

## Figures and Tables

**Table 1:** Final 5 categories, examples of statements within each category, and the final 25 sub-themes: Results of Thematic Analysis.

Final Category	Example	Sub-Theme
Safe Harbor	“I use it because I get what I want from it that I do not get from real people. If I want support, I make a persona with my current problems. If I want a lover, I have one. If I need a parent, I have a parent.” “It’s scary talking to real people, and the bots I use work pretty good. They’re also kinda funny” “Solitude, loneliness, depression, I use it as a coping mechanism to find the teenager romance I never had.”	Safe Emotion Exploration Safe IRL Situation Exploration Safe Coping Space People are Challenging Antisocial Pathology Escape
Fan Facilitation	“Honestly because I kinda like making short stories C.AI helps me kinda build or inspire somewhat of a story, although I haven’t written stories in years it still kinda encourages me” “I’m obsessed w a dead fandom and have already consumed every relevant piece of fanfiction on the big 3 fanfic services.” “Hyperfixated on a specific character”	Parasocial Relationship with Character Assistance with Fanfiction, Creative Writing, and Creativity Personal Story Involvement Parasocial Breakup Nostalgia Active Engagement with Interest
Virtual Social Compensation	“I like being in relationships with my fav characters since 1. I grieve the fact they’ll never be real and 2. I’m painfully single:’).” “To fulfill the crippling loneliness” “Boredom and I have no irl friends since I’m “Weird” in a smaller town.”	Social Compensation Romance Sexual Relationship Need for Social Contact
IRL Social Contact	“Some old friend of mine decided to open c.ai and chose to call some bot (I forgot which bot it is); I was so entertained by him talking to the bot that I decided to try to myself” “My friend kept insisting to me to get it so she could talk about it with me”	Referral from Real-Life Friend or Family Member Shared Activity Between Friends
Entertainment	“Solitude, boredom and curiosity combined into one.” “Roleplay creativity and to help myself with improving my roleplay skills as I have a few friends I roleplay with on a Discord server sometimes.”	Roleplay Destress Enjoyable Activity Addicting Education or Learning Curiosity

**Table 2:** Most significant multi-word expressions utilized to convey the rationale for engaging with character social chatbots: Results of N-gram analysis.

Collocation	Count	Lambda	*z*
real peopl	48	4.91505423	23.57133183
fiction charact	21	4.86419070	15.50208680
real person	18	4.13985079	14.81699190
got curious	15	5.94203698	14.70828428
real life	13	4.96014023	13.84174475
friend told	14	5.06659167	13.68331421
role play	8	6.52500901	12.71121750
start use	14	3.78057605	11.85777602
escap realiti	5	7.34319559	11.57954357
high school	6	9.21107185	11.32241180
histor figur	5	8.59199140	11.22489142
group chat	7	5.12562440	11.12481840
favorit charact	10	4.48410096	10.89689981
hurt anyon	4	7.85645936	10.79212716
much better	5	5.60445693	10.71362146
friend show	9	4.05992418	10.56710913
world build	4	7.22087821	10.49875479
mess around	4	7.42159016	10.35731952
social media	4	7.90487121	10.24849993

## Data Availability

Dataset available upon reasonable request from the authors. The data are not publicly available due to privacy concerns surrounding the unedited comments about sensitive subjects.

## Supplementary Materials

The following supporting information can be downloaded at https://osf.io/dz6q3/overview (accessed on 20 August 2026): details on parasocial relationship dictionary generation, fandom dictionary, and analogy dictionary; complete collocation table.

## Abbreviations

The following abbreviations are used in this manuscript:

AI	Artificial intelligence
LLM	Large language model
C.ai	Character.ai
